# Implementation of Machine Learning-Based System for Early Diagnosis of Feline Mammary Carcinomas through Blood Metabolite Profiling

**DOI:** 10.3390/metabo14090501

**Published:** 2024-09-17

**Authors:** Vidhi Kulkarni, Igor F. Tsigelny, Valentina L. Kouznetsova

**Affiliations:** 1 REHS Program, San Diego Supercomputer Center, University of California, San Diego, CA 92093, USA; vidhi.kulkarni@gmail.com; 2 CureScience, San Diego, CA 92121, USA; vkouznetsova@ucsd.edu; 3 San Diego Supercomputer Center, University of California, San Diego, CA 92093, USA; 4 Department of Neurosciences, University of California, San Diego, CA 92093, USA; 5 BiAna, La Jolla, CA 92038, USA

**Keywords:** feline mammary cancer, machine learning, metabolomics, early diagnosis

## Abstract

***Background:*** Feline mammary carcinoma (FMC) is a prevalent and fatal carcinoma that predominantly affects unspayed female cats. FMC is the third most common carcinoma in cats but is still underrepresented in research. Current diagnosis methods include physical examinations, imaging tests, and fine-needle aspiration. The diagnosis through these methods is sometimes delayed and unreliable, leading to increased chances of mortality. ***Objectives:*** The objective of this study was to identify the biomarkers, including blood metabolites and genes, related to feline mammary carcinoma, study their relationships, and develop a machine learning (ML) model for the early diagnosis of the disease. ***Methods:*** We analyzed the blood metabolites of felines with mammary carcinoma using the pathway analysis feature in MetaboAnalyst software, v. 5.0. We utilized machine-learning (ML) methods to recognize FMC using the blood metabolites of sick patients. ***Results:*** The metabolic pathways that were elucidated to be associated with this disease include alanine, aspartate and glutamate metabolism, Glutamine and glutamate metabolism, Arginine biosynthesis, and Glycerophospholipid metabolism. Furthermore, we also elucidated several genes that play a significant role in the development of FMC, such as *ERBB2*, *PDGFA*, *EGFR*, *FLT4*, *ERBB3*, *FIGF*, *PDGFC*, *PDGFB* through STRINGdb, a database of known and predicted protein-protein interactions, and MetaboAnalyst 5.0. The best-performing ML model was able to predict metabolite class with an accuracy of 85.11%. ***Conclusion:*** Our findings demonstrate that the identification of the biomarkers associated with FMC and the affected metabolic pathways can aid in the early diagnosis of feline mammary carcinoma.

## 1. Introduction

Feline mammary cancer is a type of cancer that affects the mammary glands of cats. It is one of the most common types of cancer in cats, particularly in those that have not been spayed. Mammary cancer usually begins as a small lump or nodule in one of the mammary glands and can spread to other parts of the body if left untreated. Although it is the third most common carcinoma in cats, feline mammary carcinoma (FMC) is a significantly understudied disorder. Millions of cats each year die from cancer, and early diagnosis is essential for increased survival rates. Technology, specifically machine learning (ML), may be a useful tool in the efficient, accurate, and timely diagnosis of cancer. In addition, analyzing the pathways, genes, and metabolites that play a part in the disorder can greatly develop the current understanding of FMC and assist with early diagnosis methods. The early detection and diagnosis of feline mammary cancer are crucial for a positive outcome. Recent research has focused on identifying novel biomarkers and early diagnostic tools for feline mammary cancer, including blood metabolites.

Researchers aimed to identify potential serum metabolite biomarkers for feline mammary carcinomas using liquid chromatography-mass spectrometry (LC-MS) and multiple reaction monitoring (MRM) techniques [1]. The results of the study identified 12 metabolites that were significantly different in cats with mammary carcinoma, including lysophosphatidylcholines, sphingomyelins, and Glycerophospholipids. The identified metabolites were involved in various metabolic pathways, including Glycerophospholipid metabolism, Sulphingolipid metabolism, and Fatty acid metabolism. The study concluded that the identified metabolites could serve as potential serum biomarkers for feline mammary carcinomas [1]. 

Gameiro and colleagues studied the molecular mechanisms of FMC development and utilized knowledge from human breast cancer research to identify diagnostic and prognostic biomarkers [2]. Their research offers insights into potential therapeutic options for specific subtypes of FMC, such as HER2-positive and triple-negative FMC. This study highlights the importance of using cats as a cancer model to advance our understanding of FMC and develop effective treatments [2].

The broad study of Wei and co-authors reveals that metabolites obtained using untargeted and targeted metabolomics allow to distinguish different stages of breast cancer in women [3]. Fifteen targeted metabolites with a fold change of 0.54–2.20 and *p*-value of < 0.025 and thirty-three untargeted metabolites with a fold change of 0.46–2.24 and *p*-value of < 0.049 were revealed [3]. 

Yu and colleagues identified canine mammary tumor-associated metabolites using untargeted metabolomics [4]. The study revealed 536 differential metabolites that were analyzed with MetaboAnalyst 4.0 and showed the most significant pathways: Purine metabolism, Alpha-linolenic acid metabolism, Vitamin B6 metabolism, and Fatty acid biosynthesis [4]. 

The abovementioned studies demonstrate that various blood-based biomarkers, including microRNA, metabolite, and protein biomarkers, have the potential to serve as early diagnostic tools for feline mammary cancer, which is essential for the development of new diagnostic and therapeutic strategies, which might help improve the prognosis and survival rate of affected cats. Further research is necessary to validate these biomarkers and develop practical clinical applications. Early detection and treatment of feline mammary cancer remain critical to the successful management of this disease. Although there have been studies on feline mammary cancer in the past, there is a lack of specific studies that address the metabolites that play a role in the cancer’s progression and identify machine-learning techniques that can be applied to the early diagnosis of the disorder. This highlights the novelty of our work.

## 2. Methods

### 2.1. Data Collection and Preprocessing

The feline carcinoma-related metabolites were collected from the open source data published by Zheng and co-authors [1] and are presented in Table 1. The dataset includes six female Chinese Pastoral cats with mammary gland carcinoma (positive samples) and six female Chinese Pastoral cats who were healthy samples in the data (negative samples). Before tumor removal, blood samples were retrieved from each of the cats, and metabolites were extracted from the samples. Liquid chromatography and mass spectrometry were performed, and the data were processed. In our study, the metabolites were selected when they had a *p*-value less than 0.05 and a fold change value over 1.5. The HMDB was then used to choose random metabolites and to obtain their SMILES (Simplified Molecular-Input Line-Entry System) [5,6]. The variable importance of projection (VIP) values assesses the importance of each metabolite to FMC. The randomly selected metabolites were obtained from HMDB using a random number generator without repetitions and are labeled with the corresponding label “random” in the rightmost column. 

To analyze mammary carcinoma metabolic pathways in combination with genes, the latter were extracted from two studies [7,8] and are presented in Table 2. 

### 2.2. Programs Used 

All research was completed in silico. The programs, tools, and websites used are as follows: PubChem, v. 2023 (National Center for Biotechnology Information (NCBI), Bethesda, MD, USA) [9] Human Metabolome Database (HMDB), v. 5.0 (University of Alberta, Edmonton, AB, Canada) [10], Pharmaceutical Data Exploration Laboratory (PaDEL) PaDel-Descriptor, v. 2.21 (Institute of Chemical and Engineering Sciences (ICES), Agency for Science, Technology and Research (A*STAR), Jurong Island, Singapore) [11], Waikato Environment for Knowledge Analysis (WEKA), v. 3.8.6 (University of Waikato, Hamilton, New Zealand) [12], MetaboAnalyst 5.0 (McGill University, Department of Epidemiology, Biostatistics, and Occupational Health, Montreal, QC, Canada) [13], Search Tool for the Retrieval of Interacting Genes/Proteins (STRING), v. 12.0 (Institute of Molecular Life Sciences, Zurich, Switzerland) [14], and Database for Annotation, Visualization and Integrated Discovery (DAVID), version 6.8, (Laboratory of Human Retrovirology and Immunoinformatics (LHRI), Frederick National Laboratory for Cancer Research, Frederick, MD, USA) [15].

#### 2.2.1. PaDEL-Descriptor

PaDEL-Descriptor software was employed to calculate the molecular descriptors for the selected metabolites [11]. Specifically, compounds’ SMILES were input into PaDEL-Descriptor, version 2.21, which then generated a comprehensive set of 1D, 2D, and 3D descriptors for each compound (1875 descriptors). These descriptors provide valuable information about the physicochemical and structural characteristics of the molecules, such as their size, shape, and functional groups. The use of PaDEL-Descriptor allowed for a detailed characterization of the molecular properties of the metabolites, providing a solid foundation for further analysis and interpretation of the data. In our research, we used the 1D and 2D descriptors (1444 descriptors) [16].

#### 2.2.2. Waikato Environment for Knowledge Analysis

Waikato Environment for Knowledge Analysis (WEKA), version 3.8.6, is a cutting-edge software tool that has been developed for data mining and ML [12]. We applied it to the analysis of metabolite data, where it enables efficient processing and analysis of large datasets. WEKA was utilized to perform classification tasks on a dataset using different classification algorithms. These metabolites were initially labeled as “selected” or “random” based on their source. The InfoGainAttributeEval application in WEKA was then used to evaluate the relevance of each attribute to the class label. This process effectively eliminated any unnecessary data, resulting in the selection of only significant attributes. Furthermore, WEKA’s preprocessing algorithms proved invaluable in filtering out unwanted instances through its attribute filter function. This ensured that the final dataset used for classification was optimized and devoid of any redundant information. 

#### 2.2.3. MetaboAnalyst

MetaboAnalyst 5.0, a web-based tool, has emerged as a resource for metabolomics data analysis [13]. In this study, the pathway analysis function offered by MetaboAnalyst was utilized to gain insights into the biological pathways that are potentially influenced by the 40-metabolite dataset under investigation.

The Joint Pathway Analysis application in MetaboAnalyst allows for the identification of significant pathways that may be associated with the feline mammary cancer metabolite and gene data. This function employs advanced statistical algorithms and pathway databases to identify pathways that are potentially altered or expressed in the dataset. By leveraging this feature, we were able to gain a comprehensive understanding of the metabolic pathways that are most relevant to our data.

The Network Analysis application allows one to download the sets of metabolites and genes and then visually explore these molecules’ participation in biological networks created based on known associations between genes, metabolites, and diseases [13]. 

#### 2.2.4. STRINGdb

STRING (Search Tool for the Retrieval of Interacting Genes/Proteins), version 12.0, is a biological database and web resource of known and predicted protein-protein interactions [14]. The STRING database uses information from experiments, computational predictions, and publications. The latest version, 12.0, contains information on more than 59 million proteins [14]. The tool creates network of connected proteins; connectivity (called *degree*, κ) is a measure of how centrally positioned a protein is within the interaction network; it is an important concept because highly connected proteins are often involved in key biological functions, and their dysfunction can have widespread effects on cellular systems [17].

#### 2.2.5. DAVID

DAVID (Database for Annotation, Visualization, and Integrated Discovery), version 6.8, is a bioinformatics tool that provides functional analysis of large gene lists [15]. It integrates a wide variety of biological annotations, including gene ontology, pathways, and disease associations, to help uncover the biological meaning behind gene or protein datasets. DAVID offers enrichment analysis, gene functional classification, and visualizes associations within input gene lists [15]. 

### 2.3. Data Selection and Preprocessing

Twenty metabolites were extracted from the study of Zheng and colleagues [1]. The metabolites related to FMC [1] and their corresponding values are shown in Table 1. Twenty random metabolites were obtained from HMDB using a random-number generator without repetitions. The metabolites were marked “Selected” or “Random” and compiled in the dataset, which ran through PaDEL-Descriptor, creating 1444 1D, 2D, and fingerprint descriptors. The obtained dataset with attributes applied to the WEKA’s InfoGainAttributeEval application, which reduced descriptor dimension to 31 most significant descriptors. 

For more detailed analysis, we collected additional FMC-related genes from open source studies [7,8]. 

## 3. Results

### 3.1. Metabolic Pathway Analysis

**Figure 1:**
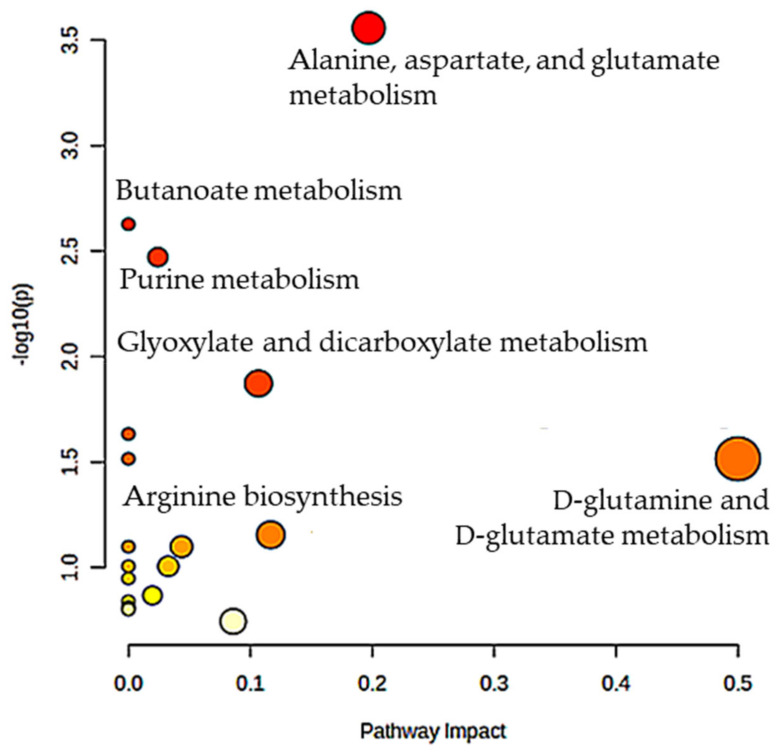
Significant metabolic pathways in FMC that were elucidated through the Metabolic Pathway Analysis application conducted using MetaboAnalyst 5.0. The analysis was performed on metabolites that play a role in FMC. The position of the pathways on the *Y*-axis and the vibrancy of the color are determined by their respective *p*-values. A higher value on the *Y*-axis and a darker shade of red color indicates greater significance of the pathway in relation to the metabolites. The size of circles indicates the number of the selected FMC metabolites in the pathways: the greater the size, the more metabolites included in pathway.

We analyzed the metabolites selected as biomarkers for FMC to support a hypothesis that these are significantly related to the cancer development process. Below, we show the pathways in which these metabolites are involved. The list of metabolites was inputted to MetaboAnalyst, v. 5.0, and the program analyzed the pathways corresponding to the metabolites submitted. The most relevant pathways had larger points on the graph (Figure 1). Below, we discuss the relation to cancer of these pathways involved.

### 3.2. Genetic Analysis Using STRING

**Figure 2:**
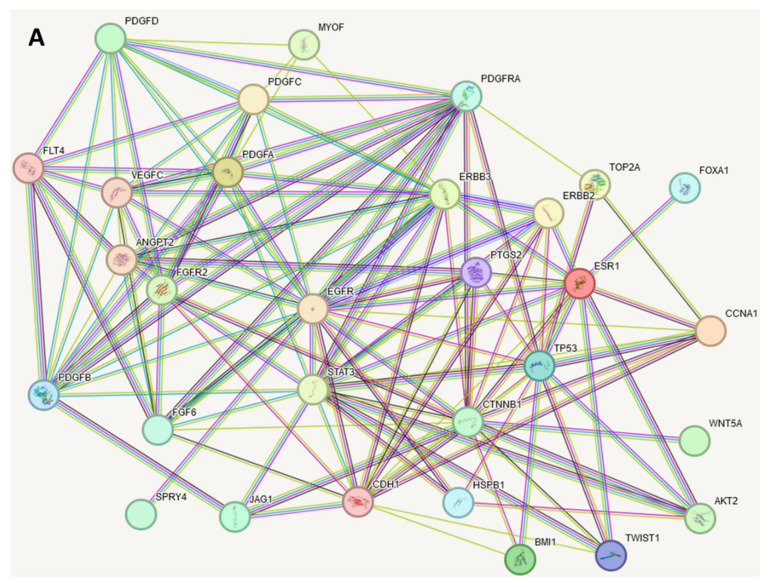

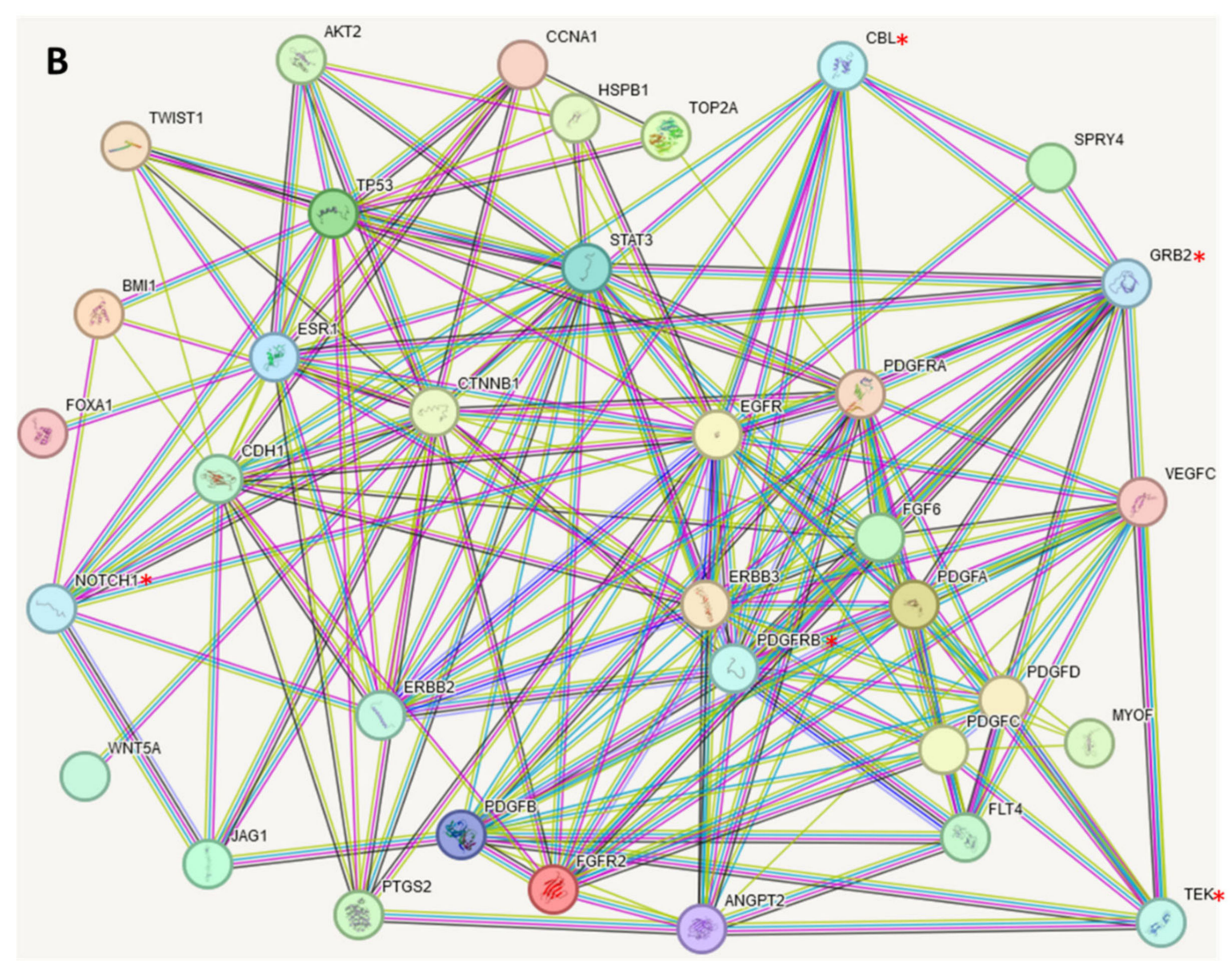
STRINGdb analyzed the set of FMC-related proteins and displayed connections between them based on their relationship to one another. Colored nodes mean query proteins and first shell of interactors; filled nodes mean that 3D structure is known or predicted; empty nodes are those proteins of unknown 3D structure. Edges are drawn with up to seven differently colored lines, which represent the existence of the seven types of evidence used in predicting the associations: red line indicates the presence of fusion evidence; green line—neighborhood evidence; blue line—co-occurrence evidence; purple line—experimental evidence; yellow line—text-mining evidence; light-blue line—database evidence; black line—co-expression evidence. (**A**) Network based on proteins corresponding to the genes in Table 2. (**B**) Enriched network with CBL, GRB2, NOTCH1, PDGFRB, and TEK proteins added (notified with the red asterisks).

Significant genes in FMC shown in Table 2 [7,8] and corresponding proteins were analyzed with the STRINGdb program, and their relationships were elucidated (Figure 2A). There were no specific criteria applied to these genes besides their discovery as significant to feline mammary carcinoma through the studies cited.

**Figure 3:**
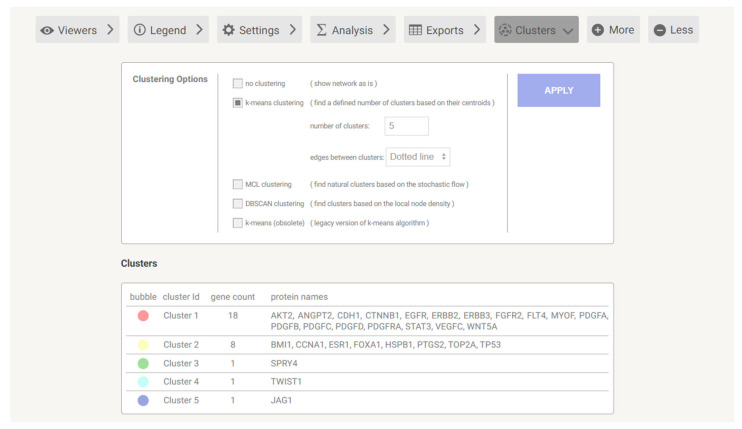
The above figure depicts the clusters created for the genes in Table 2. Each cluster was created using k-means clustering, and each cluster was analyzed through the DAVID program.

The high connectivity of the network confirms that the proteins and metabolites play a significant role in the same pathways and interact with each other. The proteins with the highest connectivity (degree, κ) are EGFR (κ = 18); STAT3 and CTNNB1 (κ = 17); CDH1, ERBB3, and p53 (κ = 14); PDGFRA (κ = 13). The group of four platelet-derived growth factors (PDGFA, PDGFB, PDGFC, and PDGFD) and VEGFC (average connectivity κ_avg_ = 10.4) have a close relationship with each other and have a large impact on the disorder (Figure 3, bottom). In FMC, these proteins encoded by appropriate genes play a role in tumor growth, invasion, and metastasis. ERBB2 (also known as HER2) is a receptor tyrosine kinase that is often overexpressed in breast cancer and has been implicated in the development of FMC as well [18]. PDGFA, PDGFB, PDGFC, and PDGFD are ligands for the platelet-derived growth factor receptors (PDGFRA and PDGFRB), which are involved in cell proliferation, migration, and survival [19]. EGFR (epidermal growth factor receptor) and ERBB3 (also known as HER3) are receptor tyrosine kinases that have been implicated in the development of cancer [20]. FLT4 (also known as VEGFR3) is a receptor for vascular endothelial growth factors (VEGFC and VEGFD), which are involved in lymphangiogenesis (the formation of new lymphatic vessels) [21]. FIGF (also known as VEGFD) is a member of the VEGF family that has been implicated in tumor growth and lymphangiogenesis [22]. CDH1 (cadherin 1) plays an essential role in epithelial cell adherence. The CDH1 mutation puts females at risk for a certain form of breast cancer called lobular breast cancer [18]. Together, *ERBB2* and *CDH1* mutations show a significantly worse prognosis for patients compared to their counterparts without such a mutation [18]. In feline mammary cancer, these genes may be dysregulated, leading to abnormal activation of signaling pathways that promote tumor growth and metastasis [7,8].

Using enrichment analysis, an additional five proteins were elucidated by STRINGdb—CBL, GRB2, NOTCH1, PDGFRB, and TEK (Figure 2B). The network presented in Figure 2A shows high-evidence associations of the proteins coded by selected genes. The additional proteins are also highly associated with the presented network (Figure 2B). The average connection association with added proteins increased from κ = 7.40 to κ = 10.46. Such an increase demonstrates that the added genes play a significant role in the organization of a strongly connected protein network involved in cancer development. This fact points to mentioned proteins as preferable targets for drug design.

CBL is a proto-oncogene that encodes a RING (interesting new gene) finger E3 ubiquitin ligase. This gene has been found to be mutated or translocated in many cancers, including acute myeloid leukemia [23].

The GRB2 is a Growth Factor Receptor Bound Protein 2. Its activation through some specific RTKs (receptor tyrosine kinase), in our case PDGFRB—class III, VEGFR3 (FLT4)—class IV, FGFR2—class V, and TEK—class IX, certifies its involvement in breast cancer progression, and its abnormal activation by ERBB2 is related with the development of human breast cancers and mammary carcinoma [21] (Figure 2B).

NOTCH1 is one of four known genes encoding the NOTCH family of proteins, a group of receptors involved in the Notch signaling pathway. Increased expression of Notch receptors has been observed in a variety of cancer types, including cervical, colon, head and neck, lung, renal, pancreatic, leukemia, and breast cancer. Activation of Notch1, among other Notch family proteins, has been shown to cause mammary carcinomas in mice [24,25].

PDGFRs (platelet-derived growth factor receptors) are catalytic receptors that have intracellular tyrosine kinase activity. Its activation induced epithelial-to-mesenchymal transition and led to Basal-like breast cancers [18]. 

TEK (Tie2) is one of the RTKs that belongs to the TIE family and, along with VEGFR, encodes angiogenesis in cancer patients [26].

The mentioned proteins participate in 75 pathways, with 22 of them directly involved in cancers (Table 3).

### 3.3. Gene Cluster Analysis through DAVID

Using STRINGdb, we performed k-means clustering on the genes from Table 2 in order to create five separate clusters with genes that are interacting with one another. K-means clustering utilizes a machine-learning model to divide a dataset into several such unique clusters. The clusters are shown in Figure 3. Submitting the genes from each cluster to DAVID (Database for Annotation, Visualization and Integrated Discovery), version 6.8 [15] we were able to identify the top pathways for each cluster. We found that the UniProt signaling pathway had the greatest number of genes from the first cluster, playing an important role in the pathway. This signaling pathway allows for the ribosome to effectively create proteins. The most prominent pathway in the second cluster was the UniProt nucleus pathway, which works within the nucleus and specifically with the pore complex. In the third cluster, the *SPRY4* gene encodes a protein known as sprouty RTK signaling antagonist 4. This protein is part of the sprouty family, which acts as inhibitors of receptor tyrosine kinase (RTK) signaling pathways. These pathways are crucial for cell growth, differentiation, and survival. In the fourth cluster, the *TWIST1* gene encodes the TWIST1 protein, which is a basic helix-loop-helix (bHLH) transcription factor. This protein plays a crucial role in various biological processes, including embryonic development, cell differentiation, and the epithelial-mesenchymal transition (EMT). For the fifth cluster, the *JAG1* gene encodes Jagged1, a ligand for the Notch receptor. Jagged1 plays a crucial role in the Notch signaling pathway, which is essential for various cellular processes, including cell fate determination, differentiation, proliferation, and apoptosis.

### 3.4. Network Analysis Using MetaboAnalyst

To show that the explored genes presented in Table 2 interact closely with the metabolites from the original open source dataset^1^ and impact mammalian breast cancer, we used MetaboAnalyst’s 5.0 Network Analysis, especially its Metabolite-Gene-Disease Interaction Network application. This network provides a scheme of potential functional relationships between metabolites, connected genes, and target diseases. Six subnetworks were elucidated: one with 86 nodes, 100 edges, and 21 seeds and five with 3 nodes, 2 edges, and 1 seed each. The main subnetwork is presented in Figure 3. The network shows that most selected metabolites and genes are connected and lead to different diseases, including cancers, with breast cancer among them. 

The breast cancer node has connectivity degree 2 and betweenness centrality 72.02. The two nodes to which it connected are CDH1 and TP53 with connectivity degrees 4 and 12 and betweenness centrality 306.39 and 1052.70, accordingly. The higher is connectivity of a node, the more significant it is. Centrality is used to measure the importance of various nodes in a network. Betweenness centrality defines and measures the importance of a node in a network based on how many times it occurs in the shortest path between all pairs of nodes in the network. Nodes with a high betweenness centrality can represent important proteins in signaling pathways and can form targets for drug discovery [27]. The network helps to elucidate the large number of genes and metabolites that impact this disease.

Genes shown in the network (Figure 3, bottom) also participate in the protein networks obtained with STRINGdb. This fact demonstrates that many of the metabolites used in our analysis are part of a large network, including several cancer-related genes. So, these metabolites are not only biomarkers but also participants in the cancer development process.

### 3.5. Joint Pathway Analysis Using MetaboAnalyst

**Figure 4:**
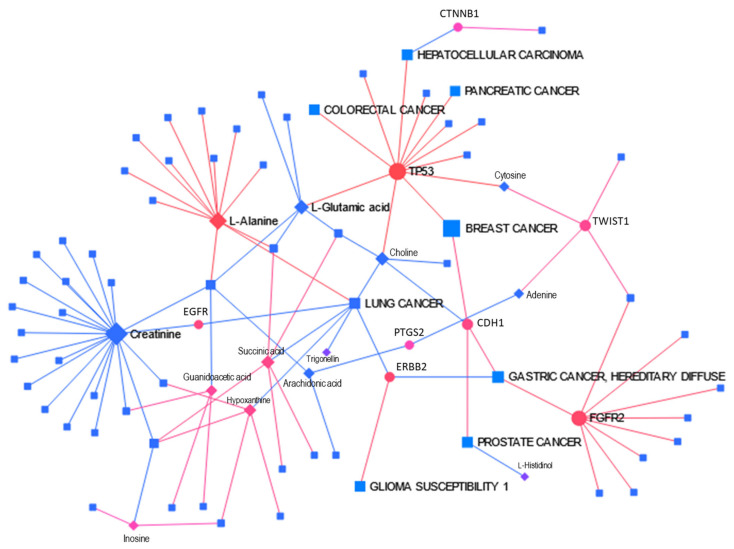
Metabolite-Gene-Disease Interaction Network created through MetaboAnalyst’s 5.0 Network Analysis application. A total of 35 genes and 21 metabolites were analyzed. Six subnetworks were created: subnetwork 1 with 86 nodes, 100 edges, and 21 seeds and five subnetworks with 3 nodes, 2 edges, and 1 seed. Subnetwork 1 is more informative. It contains 8 genes and 13 metabolites. Shapes represent the following: circles—genes, diamonds—metabolites, and squares—diseases; colors show the following: red—activated, blue—inhibited, and purple—neutral; size represents importance. Edge color represents correlation: red—positive; blue—negative.

To study metabolic and signaling pathways in mammalian breast cancer, we used MetaboAnalyst’s 5.0 Joint Pathway Analysis application, choosing All Integrated Pathways function with both inputs—metabolites and genes. The results of this analysis are shown in Figure 4.

The significance of the pathway is determined by *p*-value and impact. The lower the *p*-value, the more significant the pathway. Pathway impact represents a combination of centrality and pathway enrichment results. Centrality assigns importance to nodes in the pathway. To calculate the impact, we used the degree centrality and the hypergeometric test. Higher impact values represent the relative importance of the pathway. 

The MAPK signaling pathway, Pathways in cancer, and EGFR tyrosine kinase inhibitor resistance have the best *p*-values: 7.823 × 10^−19^, 9.137 × 10^−19^, and 3.891 × 10^−18^, correspondingly. The breast cancer pathway has *p* value = 3.608 × 10^−13^ and an impact score of 0.45, which reflects that this pathway has an over-average significance. 

MAPK signaling pathway plays an important role in cancer development because its abnormal activation leads to increased/uncontrolled cell proliferation and resistance to apoptosis. Its activation is responsible for around 40% of all cancers [28]. The Cancer pathway includes 14 significant pathways whose functions are necessary for cancer growth and progression [29].

The JAK-STAT signaling pathway is involved in the proliferation, progression, metastasis, and survival of various types of tumor cells. It plays an important role in mammary cancer development [30].

The ErbB signaling pathway regulates cell proliferation, migration, differentiation, apoptosis, and cell motility. It includes four family members: ERBB1 (EGFR, or HER1), ERBB2 (HER2), ERBB3 (HER3), and ERBB4 (HER4). They are over-expressed, amplified, or mutated in many forms of cancer, especially in breast cancer [20].

**Figure 5:**
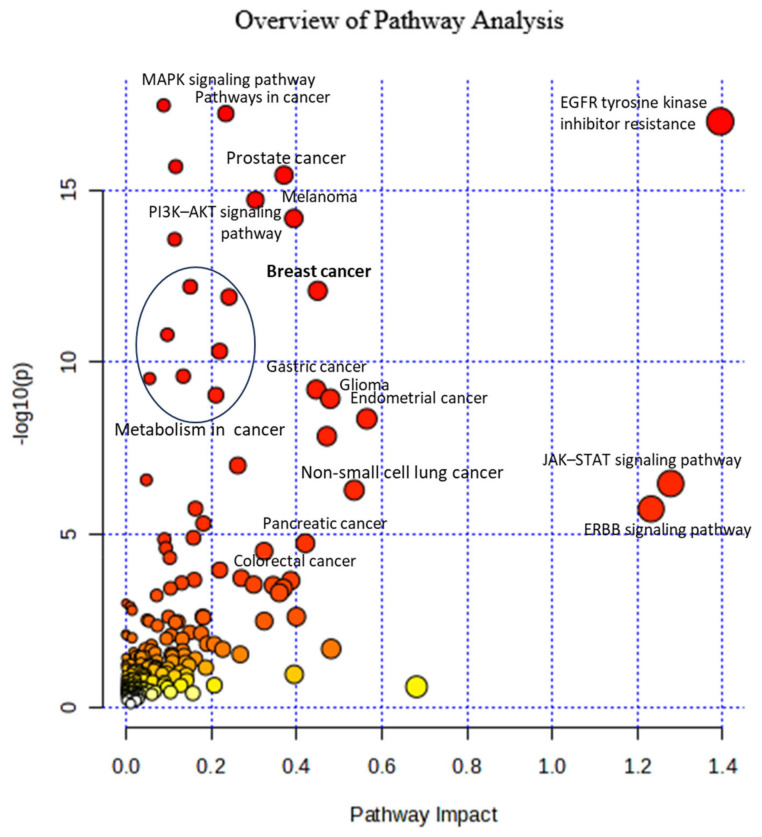
Integrated Pathway Analysis of metabolite and gene biomarkers shown in breast cancer. The position of the pathways on the *Y*-axis is determined by their respective *p*-values. The larger the size of the circle, the greater the pathway enrichment. The darker the color of each circle on the plot, the greater its significance. The size of circles indicates the ratio of number of elements involved in pathway to the number of all pathway’s members. The vibrancy of the color reflects pathway impact score, which represents significance of given pathway relative to global integrative network. Outlined by oval, the following are metabolic pathways important in cancer development: Choline metabolism, Focal adhesion, Central carbon metabolism, Proteoglycans in cancer, Phospholipase D signaling pathway, and GAP junction.

There are also some metabolism pathways: Choline metabolism, Focal adhesion, Central carbon metabolism, Proteoglycans in cancer, Phospholipase D signaling pathway, and GAP junction, which are important in cancer development (outlined by the oval in Figure 5).

### 3.6. Machine Learning Classifiers

ML has gained popularity in cancer studies and biomedical data analysis for identifying patterns. In our study, we used a final dataset comprising 40 metabolites, with 20 selected from a previous study [1] and 20 randomly selected from an HMDB. The filtered dataset consisted of 31 attributes. We employed various classifiers, such as SGD, Random Forest, Hoeffding Tree, and others, to evaluate the accuracy of classification.

**Figure 6:**
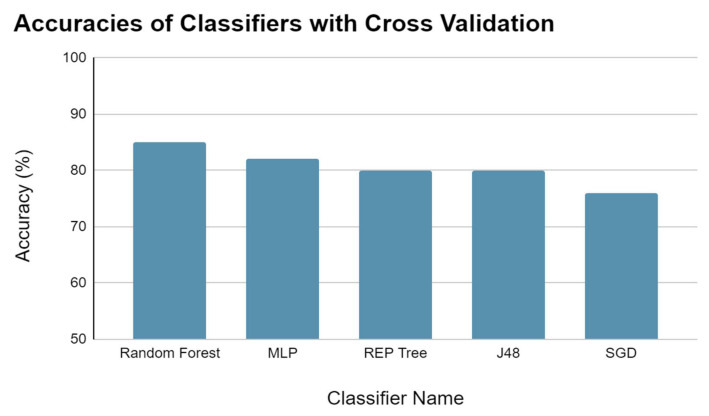
The classifier performances with different algorithms obtained through cross-validation classifier.

Among the classifiers, J48, REP Tree, MLP, and Random Forest showed accuracy above 80%, with Random Forest achieving the highest accuracy of 90.02% (Figure 6). To evaluate the classifiers, we used cross-validation, which involves dividing the dataset into ten folds and testing each fold on the remaining nine folds for training, repeating the process ten times for each distinct fold. The results of each test were then averaged to obtain the accuracy of each classifier. Cross-validation helps in reducing the variance of the estimate, making the results more reliable. 

**Figure 7:**
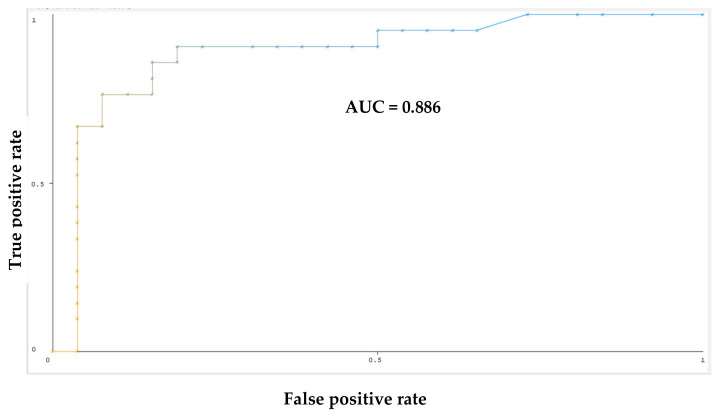
ROC curve for Random Forest Model. The ROC (receiver operating characteristic) curve is a graphical representation of the performance of a classifier in distinguishing between positive and negative samples. The colors of the curve represent threshold value set to get the best pair of true FPR/TPR point.

Figure 7 shows the ROC (receiver operating characteristic) curve for the ML classifier. The *X*-axis of the curve plots the false positive rate, and the *Y*-axis plots the true positive rate. This curve reflects the performance of the model at various classification thresholds. The comparison of these values through the curve allows for the identification of how likely the model is to maximize true positives and minimize false positives in the data. The area under the curve (AUC) is 0.886, which is close to 1. AUC provides an aggregate measure of performance across all possible classification thresholds; the higher the AUC, the better the performance. There is another characteristic to evaluate performance of ML model—the precision-recall curve, which is the measure between precision and recalls for different thresholds. As the curve approaches the top right of the graph, it demonstrates that the model has good prediction accuracy. (Table 4).

The selected performance measures of the models are presented in Table 5. The best accuracy is obtained with the Random Forest classifier, which is recommendable for implementation. More detailed measures related to the performance of the best ML models are presented in Table 4.

## 4. Discussion

### 4.1. Alanine, Aspartate, and Glutamate Metabolism

The metabolic pathways involving alanine, aspartate, and glutamate, three important amino acids, have been implicated in the development and progression of FMC. Understanding the role of these amino acids in cancer metabolism may shed light on the underlying mechanisms of this cancer [1]. 

Alanine, aspartate, and glutamate are interrelated amino acids that are involved in various cellular processes, including protein synthesis, energy production, and redox balance. The metabolism of these amino acids is tightly regulated by a group of enzymes known as aminotransferases, which facilitates the transfer of amino groups between these amino acids. Dysregulation of these enzymes and altered expression of genes involved in the metabolism of these amino acids have been observed in cancer, including FMC in cats [31].

One of the key roles of alanine metabolism in cancer is its involvement in cellular energy production. Alanine can be converted into pyruvate, a crucial intermediate in cellular respiration, through the action of the alanine aminotransferase enzyme. Pyruvate can then be utilized in the citric acid cycle to generate ATP, the primary energy currency of cells. Dysregulation of alanine metabolism has been associated with increased cell proliferation and survival in several cancers, and it may contribute to the metabolic rewiring that occurs in cancer cells to meet their increased energy demands [32]. 

Aspartate metabolism is also implicated in cancer cell proliferation and survival. Aspartate can be converted into oxaloacetate, another intermediate in the citric acid cycle, through the action of aspartate aminotransferase enzyme. Oxaloacetate can then be utilized in the biosynthesis of nucleotides, which are essential for DNA synthesis and cell division. Alterations in aspartate metabolism have been shown to impact cellular energy production, redox balance, and nucleotide synthesis, all of which are critical for cancer cell growth and survival [33].

Glutamate metabolism, on the other hand, has been linked to cancer cell invasion and metastasis. Glutamate can be converted into α-ketoglutarate, another intermediate in the citric acid cycle, through the action of glutamate dehydrogenase enzyme. α-ketoglutarate can then be utilized in the biosynthesis of collagen and other extracellular matrix components, which are crucial for cancer cell invasion and metastasis. Dysregulation of glutamate metabolism has been associated with enhanced cancer cell migration, invasion, and metastasis in various cancers [34].

Moreover, alterations in the metabolism of these amino acids may also impact the tumor microenvironment and immune response in feline mammary cancer. For example, changes in the levels of alanine, aspartate, and glutamate have been detected in the blood and tissue samples of cats with mammary cancer, suggesting that these amino acids may serve as potential biomarkers for early detection and prognosis of feline mammary cancer. Additionally, the metabolism of these amino acids has been shown to modulate the activity of immune cells in the tumor microenvironment, potentially influencing the immune response against cancer cells [1].

### 4.2. Glutamine and Glutamate Metabolism

The metabolism of glutamine and glutamate, two important amino acids, has been implicated in the development and progression of feline mammary cancer, a malignant neoplasm that occurs in the mammary glands of cats. These amino acids play critical roles in various cellular processes, including protein synthesis, energy production, and redox balance, and dysregulation of their metabolism has been associated with cancer-related pathways in several cancers, including mammary cancer in cats [1].

Glutamine is a non-essential amino acid that serves as a major source of nitrogen and carbon for cancer cells. It can be converted into α-ketoglutarate, an intermediate in the citric acid cycle, through the action of glutaminase enzyme. α-ketoglutarate can then be utilized in the biosynthesis of nucleotides, which are essential for DNA synthesis and cell division. Dysregulation of glutamine metabolism has been shown to impact cellular energy production, redox balance, and nucleotide synthesis, all of which are critical for cancer cell growth and survival [35].

Similarly, glutamate, another non-essential amino acid, plays a key role in cancer metabolism. It can be converted into α-ketoglutarate through the action of amino acid oxidase enzyme, which generates hydrogen peroxide as a byproduct. Hydrogen peroxide is a reactive oxygen species (ROS) that can cause oxidative stress and damage to cellular components, including DNA, proteins, and lipids. Dysregulation of glutamate metabolism and the accumulation of ROS have been linked to DNA damage, genomic instability, and tumor progression in various cancers [36]. 

Furthermore, alterations in the metabolism of glutamine and glutamate may also impact the tumor microenvironment and immune response in feline mammary cancer. For instance, changes in the levels of glutamine and glutamate have been detected in the blood and tissue samples of cats with mammary cancer, suggesting that these amino acids may serve as potential biomarkers for early detection and prognosis of feline mammary cancer. Additionally, the metabolism of glutamine and glutamate has been shown to modulate the activity of immune cells in the tumor microenvironment, potentially influencing the immune response against cancer cells [1].

### 4.3. Arginine Biosynthesis

The biosynthesis pathway of arginine, an essential amino acid, has been suggested to potentially play a role in feline mammary cancer, a malignant neoplasm that occurs in the mammary glands of cats. Arginine is a crucial amino acid involved in various physiological processes, including protein synthesis, wound healing, immune response, and regulation of nitric oxide production. Dysregulation of the Arginine biosynthesis pathway has been implicated in cancer development and progression in several cancers, and its potential involvement in feline mammary cancer warrants investigation [37]. 

The Arginine biosynthesis pathway involves a series of enzymatic reactions that convert citrulline, an intermediate in the urea cycle, into arginine. This pathway is critical for maintaining intracellular levels of arginine, which is required for various cellular functions. However, cancer cells often exhibit alterations in metabolic pathways to meet their increased demand for energy and building blocks for rapid cell growth, and the Arginine biosynthesis pathway may be affected in cancer cells as well [38]. 

Cancer cells may exhibit increased expression of argininosuccinate synthase (ASS1), a key enzyme in the Arginine biosynthesis pathway, leading to enhanced production of arginine within the tumor microenvironment. This can result in increased availability of arginine for cancer cells, promoting their proliferation and survival [39]. 

On the other hand, some cancers, including melanoma and hepatocellular carcinoma, have been shown to downregulate ASS1 expression, leading to arginine depletion in the tumor microenvironment. This can lead to a state of arginine auxotrophy, where cancer cells become dependent on exogenous arginine for survival. In such cases, arginine deprivation strategies have been explored as potential therapeutic approaches, as they can selectively target cancer cells that lack ASS1 expression while sparing normal cells that can produce arginine through the biosynthesis pathway [40]. 

### 4.4. Glyoxylate and Dicarboxylate Metabolism

Recent studies have shown that alterations in the Glyoxylate and dicarboxylate metabolism pathway may contribute to cancer development and progression by influencing key cellular processes. For example, the dysregulation of enzymes involved in this pathway, such as isocitrate lyase (ICL) and malate synthase (MS), has been shown to promote tumor growth and metastasis in certain types of cancer. These enzymes are involved in the conversion of glyoxylate and dicarboxylate compounds into intermediates of the tricarboxylic acid (TCA) cycle, which plays a crucial role in cellular energy production [41].

### 4.5. Purine Metabolism

Alterations in the Purine metabolism pathway may contribute to cancer development and progression by influencing key cellular processes. For example, dysregulation of enzymes involved in this pathway, such as adenosine deaminase (ADA) and purine nucleoside phosphorylase (PNP), has been shown to promote tumor growth and metastasis in certain types of cancer. These enzymes are involved in the degradation of purine compounds, and their dysregulation can lead to the accumulation of purine metabolites, which can potentially disrupt cellular processes and promote cancer cell survival and proliferation [42]. 

Moreover, the Purine metabolism pathway has been implicated in the regulation of immune response and inflammation, which can play a critical role in cancer development. Purine metabolites, such as adenosine, can modulate immune response by acting as signaling molecules that regulate immune cell function and inflammation. Dysregulation of purine metabolism can disrupt the balance of purine metabolites, leading to altered immune response and inflammation, which can promote cancer cell survival and facilitate tumor growth [43].

### 4.6. Butanoate Metabolism

Butanoate has been shown to have diverse effects on cellular processes that are implicated in cancer development. It has been reported to affect cell proliferation, apoptosis, and inflammation, which are key processes involved in cancer progression. For example, butanoate has been shown to inhibit the proliferation of cancer cells by inducing cell cycle arrest and apoptosis, which can suppress tumor growth. On the other hand, butanoate has also been reported to promote inflammation, which can contribute to cancer development and progression by stimulating angiogenesis, immune cell infiltration, and tissue remodeling [44].

## 5. Conclusions

Our hypothesis that it is possible to create an ML model to effectively diagnose early feline mammary cancer using blood metabolites was confirmed by our calculations. We developed a Random Forest-based ML model that is able to predict feline mammary carcinoma with 85.11% accuracy. In addition to the classifier, we studied the metabolic pathways related to feline mammary cancer. We analyzed the relationships between genes that interacted with the blood metabolites. With bioinformatics tools, we were able to identify that most of the metabolites used to train the ML model corresponded to the pathways that cause feline mammary carcinoma and interact with the known FMC-related genes. 

Our study shows the applicability of ML classifiers and the analysis of metabolomic pathways and genes to the veterinary sciences. Improvements to the developed model could be achieved by acquiring a larger dataset, which includes additional metabolites and descriptors. Although the impacts of our results are vast and important, it is significant to note that we call for more frequent feline cancer screening as they will not only assist in early diagnosis but they will also allow for the better application of our results and machine learning model. The limitations in available feline mammary cancer metabolite data were the primary challenge that we had to overcome and impacted how accurate our model could become. In the future, similar models could be developed for other feline carcinomas and could even be applicable to the early diagnosis of cancer in other species, including humans.

## Figures and Tables

**Table 1:** FMC-related metabolites [1].

Metabolites	*p*-Value	Increase/Decrease	Fold Change
L-glutamate	9.39957 × 10^−6^	Increase	2.301030
L-alanine	9.66197 × 10^−5^	Increase	1.903090
Glycerol 3-phosphate	1.128205 × 10^−3^	Increase	1.591065
Succinate	5.54729 × 10^−4^	Increase	1.446256
20-hydroxy-PGE2	2.17846 × 10^−5^	Increase	2.079181
Fosfomycin	2.655475 × 10^−3^	Increase	2.041393
3-methyluridine	2.77439 × 10^−4^	Increase	1.748188
N-acetyl-L-alanine	2.40845 × 10^−4^	Increase	1.698970
Choline	2.37754 × 10^−7^	Increase	1.778151
Trigonelline	2.40845 × 10^−4^	Increase	1.612784
Ile-Asn	1.10469 × 10^−5^	Increase	1.724276
Arachidonic acid (peroxide free)	1.126785 × 10^−3^	Increase	1.556303
S-methyl-5′-thioadenosine	3.60333 × 10^−4^	Increase	1.643453
Creatinine	5.23436 × 10^−5^	Increase	1.568202
L-histidinol	8.41015 × 10^−4^	Increase	1.602060
Guanidine acetic acid	5.46455 × 10^−4^	Increase	1.653213
Cytosine	1.12223 × 10^−4^	Increase	1.690196
Inosine	2.446273 × 10^−3^	Decrease	−1.522878
Adenine	1.575227 × 10^−3^	Increase	1.518514
Hypoxanthine	6.7303 × 10^−4^	Increase	1.585027
L-glutamic acid	1.94106 × 10^−2^	Increase	1.579784

**Table 2:** FMC-related genes.

FMC-Related Genes	References
*AKT2*	[8]
*ANGPT2*	[7]
*BMI1*	[8]
COX-2 (*PTGS2*)	[8]
Cyclin A1 (*CCNA1*)	[8]
E-cadherin (*CDH1*)	[8]
*EGFR*	[7,8]
*ERBB2*	[7,8]
*ERBB3*	[7]
ERalpha (*ESR1*)	[8]
FGF2 (*FGFR2*)	[7]
*FOXA1*	[8]
*HSPB1*	[7]
*JAG1*	[8]
*MYOF*	[7]
*PDGFA*	[7]
*PDGFB*	[7]
*PDGFC*	[7]
*PDGFD*	[7]
*PDGFRA*	[7]
*STAT3*	[7]
f-STK (*SPRY4*)	[7]
*TOP2A*	[8]
*TP53*	[8]
*TWIST1*	[8]
*VEGFC*	[7]
VEGFD (*FGF6*)	[7]
VEGFR3 (*FLT4*)	[7]
*WNT5A*	[8]
B-catenin (*CTNNB1*)	[8]

**Table 3:** FMC-related pathways. In bold is shown breast cancer having highest betweenness centrality of two contacting proteins CDH1 and p53 (encoded by *TP53*) (Figure 4).

Pathway	Number of Proteins	Strength	FDR
Melanoma	9 of 64	1.99	3.60 × 10^−4^
Choline metabolism in cancer	7 of 87	1.75	1.69 × 10^−9^
Endometrial cancer	4 of 52	1.73	1.76 × 10^−5^
Glioma	5 of 67	1.72	8.47 × 10^−7^
Thyroid cancer	2 of 32	1.64	7.60 × 10^−3^
Central carbon metabolism in cancer	4 of 65	1.63	3.91 × 10^−5^
Bladder cancer	2 of 35	1.60	8.30 × 10^−3^
Gastric cancer	7 of 127	1.59	1.60 × 10^−8^
**Breast cancer**	**7 of 131**	**1.57**	**1.83 × 10^−8^**
MicroRNAs in cancer	7 of 141	1.54	2.79 × 10^−8^
Non-small cell lung cancer	3 of 62	1.53	1.10 × 10^−3^
Pancreatic cancer	3 of 64	1.52	1.20 × 10^−3^
Acute myeloid leukemia	3 of 65	1.51	1.20 × 10^−3^
Proteoglycans in cancer	8 of 187	1.48	6.60 × 10^−9^
Colorectal cancer	3 of 75	1.45	1.70 × 10^−3^
PD-L1 expression and PD-1 checkpoint pathway in cancer	3 of 83	1.40	2.10 × 10^−3^
Basal cell carcinoma	2 of 55	1.40	1.81 × 10^−2^
Pathways in cancer	15 of 475	1.34	1.93 × 10^−15^
Kaposi sarcoma-associated herpesvirus infection	5 of 162	1.33	4.81 × 10^−5^
Renal cell carcinoma	2 of 66	1.33	2.38 × 10^−2^
Hepatocellular carcinoma	4 of 140	1.30	5.50 × 10^−4^
Transcriptional misregulation in cancer	3 of 154	1.13	9.10 × 10^−3^

**Table 4:** Performance measures of the best ML models.

Classifier	TP Rate	FP Rate	Precision	Recall	F-Measure	MCC	AUC	AUCPR	Class
Random F	0.857	0.154	0.818	0.857	0.837	0.701	0.886	0.819	select
Random F	0.846	0.143	0.880	0.846	0.863	0.701	0.886	0.904	random
MLP	0.810	0.154	0.810	0.810	0.810	0.656	0.846	0.800	select
MLP	0.846	0.190	0.846	0.846	0.846	0.656	0.846	0.867	random

Random F—Random Forest; MLP—Multilayer Perceptron.

**Table 5:** Selected performance measures of the ML models in cross-validation.

Parameter	Random Forest	MLP	REPTree	J48	SGD
Correctly Classified Instances	85.11%	82.98%	80.85%	80.85%	76.60%
Kappa statistic	0.70	0.65	0.61	0.61	0.53
Mean absolute error	0.25	0.19	0.27	0.27	0.23
Root mean squared error	0.35	0.39	0.40	0.40	0.48

## Data Availability

The data that support the findings of this study are available from the corresponding author upon reasonable request.
